# Staff disability data in UK higher education: Evidence from EDI reports

**DOI:** 10.1136/medhum-2024-012892

**Published:** 2024-06-04

**Authors:** Eirini-Christina Saloniki, Kristoffer Halvorsrud, Isabelle Whelan, Nishat Halim, Riya George, Chloe Orkin

**Affiliations:** 1 Primary Care and Population Health, University College London, London, UK; 2 NIHR Applied Research Collaboration North Thames, London, UK; 3 The Blizard Institute, Queen Mary University of London, London, UK; 4 Barts Health NHS Trust, London, London, UK; 5 Equality, Diversity and Inclusivity, Association for the Study of Medical Education, Edinburgh, UK

**Keywords:** Medical humanities, disability, Education

## Abstract

**Objectives:**

To explore how higher education institutions (HEIs) make transparent the data they collect on staff disability, and how this relates to existing equality, diversity and inclusion (EDI) charters.

**Design:**

Descriptive cross-sector quantitative study based on UK HEIs.

**Setting:**

Higher education sector in the UK.

**Participants:**

162 HEIs across the UK with information extracted from the Higher Education Statistics Agency (HESA), each institution’s website and Advance HE.

**Primary and secondary outcome measures:**

Availability of a publicly available EDI report. Type of information on staff disability identified within the EDI report and level of detail, the latter derived from the number of different types of information provided in the report. Athena SWAN and Disability Confident award level for each HEI were used as a proxy for the sector’s commitment to EDI.

**Results:**

Under a quarter of HEIs do not have an open EDI report online. The majority of Athena SWAN award holders make their EDI reports publicly available, which is similar by Disability Confident status. Russell Group universities are more likely to have a publicly available report. Regionally, EDI report availability is lowest in London. The level of detail with regards to staff disability varies, with more than half of institutions providing ‘little detail’ and just under a third ‘some detail’. Athena SWAN award holders and Disability Confident members are twice as likely to provide ‘some detail’ than those which do not hold an award.

**Conclusions:**

Challenges remain to obtain a clear picture of staff with disabilities within higher education. The lack of both uniformity and transparency in EDI reporting with respect to disability hinders the ability to quantify staff with disabilities within higher education, develop meaningful interventions and address inequities more widely.

## Introduction

 Attracting and retaining a rich diversity of staff in higher education with respect to protected characteristics is essential to produce inclusive innovative research, and to allow students to be educated by staff that represent, reflect and can be role models of students’ intersectional identities and the communities in which they are embedded. In the UK, the Equality Act 2010 enshrines the principles of advancing equality of opportunity and fostering good relations for a range of protected characteristics including but not limited to disability, sex, gender identity, race, age, pregnancy status and sexual orientation (HM Government 2010). With respect to disability, the Equality Act places a duty on employers to make ‘reasonable adjustments’ in order to eliminate unlawful discrimination, harassment and victimisation.

Additionally, higher education institutions (HEIs) have a formal duty to collect and report on equalities monitoring data to fulfil their public sector equality duty (PSED; HM Government 2011). Similar provisions apply to HEIs in Northern Ireland through section 75 of the Northern Ireland Act 1998. How institutions interpret this duty varies (Baltaru 2019). Many HEIs have equality, diversity and inclusion (EDI) officers and make institutional policies and monitoring data publicly available. However, research has shown that many policies are not assessed for EDI impact nor do they elaborate on the actions that will be taken to achieve equity and inclusion, with many institutions approaching EDI from a managerial or legalistic perspective (Koutsouris, Stentiford, and Norwich 2022).

Over the past 20 years, a number of frameworks have been introduced to accredit institutions for their efforts to address inequities, such as the Athena SWAN Charter, which aims to support gender equity within higher education and research (Advance HE n.d.-a; Xiao *et al* 2020). There are similar initiatives addressing other protected characteristics, such as the Race Equality Charter (Advance HE n.d.-b) and the Stonewall Diversity Championship programme. In 2013, the Department for Work and Pensions (DWP) introduced the Disability Confident Employer Scheme, which is designed to encourage employers to recruit, retain and develop people with disabilities (Department for Work and Pensions 2016). Organisations can progress through the voluntary scheme’s levels by undertaking certain activities in terms of recruiting and enabling employees with disabilities. To achieve the higher levels, employers should undertake self-assessment, provide reporting and undergo external validation. DWP emphasises that Disability Confident employers are challenging attitudes and increasing understanding of disability as well as removing barriers and ensuring access for people with disabilities. This means access that goes beyond wheelchair ramps and also considers people, for example, with visual and hearing challenges. A number of HEIs have signed up to the scheme.

Such charter marks have been recognised as a mechanism through which to foster positive change within HEIs, revealing inequities through data reporting and action planning, and providing a framework for constructive conversations (Bhopal and Henderson 2019). However, they have also been critiqued as ‘tick box’ exercises, with concerns that ‘achieving’ an award suggests EDI work is completed, and that inclusion is treated as a performance indicator to help institutions win grant funding, attract staff and students (Bhopal and Henderson 2019; Koutsouris, Stentiford, and Norwich 2022). Adding to this, EDI work is often delivered by women and people from racially minoritised groups creating an unseen labour or ‘time tax’ which can affect their career progression, especially if citizenship roles do not form part of promotion processes and if these roles do not attract workforce time allocations or remuneration (Gewin 2020).

How disability is conceptualised and experienced varies, with two broad approaches in the literature: a biomedical ‘deficit’ model focused on individual illness and impairment, and a social model in which disability is constructed as a result of social and environmental barriers to participation (Brown and Leigh 2018). However, a social-relational model has been posited, which examines the ways in which people are affected both by their abilities and by the interactions between those abilities and the social context (Merchant *et al* 2020).

The Equality Act 2010 defines a person as having a disability if they have ‘a physical or mental impairment, and the impairment has a substantial and long-term adverse effect on his or her ability to carry out normal day-to-day activities’ (HM Government 2010). However, individuals’ own identification as having a disability may not overlap with this definition. Figures from 2020/2021 show that 6.0% of all staff working in UK HEIs declared a known disability (Advance HE 2022a). When distinguishing between academic and non-academic staff, 5.1% (7.0%) of academic (non-academic) staff disclosed a disability, compared with 5.4% of managers, directors and senior officials (Advance HE 2022a). Although these figures show an increase from 2014/2015, they are far lower than the one in five of the UK working-age population who are classed as having a disability (Department for Work and Pensions 2023).

Much of the literature on disability within higher education focuses on students rather than staff, a demarcation that is reflected in university policies and practice (Merchant *et al* 2020). In addition, the research on staff primarily focuses on academic rather than non-academic university staff, with the two groups potentially experiencing overlapping but different issues in the workplace. Staff can face issues from recruitment onwards, including finding themselves bearing the burden of making the necessary adjustments (Anderson *et al* 2023; Inckle 2018; Merchant *et al* 2020). For academic staff, disability may affect their academic identity, particularly within an ableist workplace culture which prioritises productivity and research and can stigmatise difference and perceived impairment (Brown and Leigh 2018; Dolan 2023). This can contribute to staff having reservations about declaring their disability status, especially for people with invisible disabilities, and where many staff—particularly early career researchers—are employed on precarious fixed-term contracts (Brown and Leigh 2018).

There is limited literature on the practical impact of EDI reporting or of charters such as the Disability Confident Scheme. As guidance from Advance HE notes and evidence from Australia support, data reporting in itself is necessary but not sufficient to advance equity and inclusion (Advance HE 2022b; Mellifont 2020). Wolbring and Lillywhite (2021) further note that *how* EDI interventions are delivered affects their impact for staff and students within higher education, and also how academia engages with wider issues facing people with disabilities.

Despite these caveats, EDI monitoring and reporting is an important first step which allows HEIs to identify inequities and existing barriers within their organisation, which can then feed into the design of bespoke interventions to be evaluated over time (Advance HE 2022b). EDI reporting also builds confidence in a supportive and transparent workplace culture. Indeed, staff are more likely to disclose disability if they are confident in their institution’s commitment to EDI and that such a disclosure will not affect their career progression (Brewster *et al* 2017). Conversely, HEIs that fail to seriously address EDI may be avoided by individuals with protected characteristics who believe they could be disadvantaged, including staff with disabilities.

In this paper, we address this gap in the literature by exploring *how* HEIs use the data they collect on staff disability, and how this relates to existing EDI charters, to highlight what is seen in practice and the effect of EDI reporting. We assess whether UK HEIs have a publicly available EDI report, and where these reports can be found, we analyse the level of detail provided about staff disability, and if there is any relationship between the level of detail and an institution’s Athena SWAN and Disability Confident statuses. Additionally, we look at whether there are regional differences or differences between research-intensive Russell Group universities and other universities.

## Methods

This is a descriptive study in which we assessed whether UK HEIs have a publicly available EDI report, whether this includes information on staff disability and its level of detail in this regard.

### Data collection

First, we obtained a list of all UK HEIs (n=213) via the experts in UK higher education data and designated data body for England—the Higher Education Statistics Agency (Higher Education Statistics Agency (HESA) 2021a). Second, we manually searched for the presence of EDI report(s) for each HEI via the respective institution’s website. Third, for each institution we extracted data from the latest available report only (in Scotland the requirement for publication can be less frequent at up to 2 years) (HM Government 2012). The cut-off date for the report searches was 10 June 2022. If no links to reports were identified, or reports were only available to access internally via log-in for staff working at the specific institution, or did not contain relevant data on staff disability, we deemed these institutions to have no publicly available reports on staff disability.

All data fields and descriptions of the types of information we extracted from the reports and other verified sources can be found in online supplemental table 1. They covered, for example, information about staff disability type, disability prevalence by faculty/service area, staff group, contract type and grade. We further identified via HESA whether the institution opted out of returning information about non-academic staff (this became an option from 2019/2020; Higher Education Statistics Agency (HESA) 2021a), whether the HEI belonged to the Russell Group (ie, group of 24 UK universities with a shared focus on research and reputation for academic achievement), and if they were a member of different EDI charters—the Athena SWAN Charter and Disability Confident Employer Scheme, as these were most relevant and frequently mentioned in HEIs’ EDI missions. The charters could also be related to, more broadly, the commitment of the sector to EDI. The report year and disability status were the most challenging fields to categorise as the recording was largely inconsistent over time and across institutions (see notes in online supplemental table 1).

### Data analysis

For each publicly available EDI report, we assessed the level of detail on staff disability, defined as whether (on top of current staff disability prevalence) it included information on any of the following fields: disability prevalence trends, disability type, faculty/service area, staff group, contract type, employment mode and grade. An aggregate score was subsequently calculated per institution, with total scores varying between 0 and 7. To allow meaningful comparisons, the aggregate scores were grouped into three categories: 0 (no detail; 0 fields), 1 (little detail; 1–2 fields), 2 (some detail; more than 2 fields). Further, we examined whether there were differences in report availability by institution type (ie, Russell vs non-Russell Group), geography (ie, region), and whether institutions opted out of returning information about non-academic staff in 2019/2020 onwards. Due to variation in institution size and potential correlation with the regional analysis, we further considered staff size. Staff size included all staff except for atypical staff (as for the latter only a minimum data set is required), obtained by report year where possible (Higher Education Statistics Agency (HESA) 2021b). Staff size for institutions with EDI reports prior to 2014/2015 (n=1) and in 2021/2022 (n=15) was not available, thus these institutions were excluded from the respective analyses. For institutions with no publicly available report, the most recent staff size estimates (ie, in 2020/2021) were used to allow for comparisons. For supplementary analyses looking at variations by commitment to a specific EDI charter, we also explored the impact of the membership level, where possible (ie, Athena SWAN: Bronze, Silver, Gold; Disability Confident Employer: Committed (Level 1), Employer (Level 2) and Leader (Level 3)). The analysis excluded private/for-profit institutions (n=1) and those with fewer than 150 employees as these do not fall under the PSED (n=50). The final sample considered in the analysis is 162 HEIs. We used Excel for data extraction while the analysis was performed in Stata V.17.0 (StataCorp 2021). All descriptive statistics are reported as proportions or percentages, and the lower statistical significance level—for t-tests used to check for significance in variations—was set at 0.01. A set of results are also displayed in bar charts.

### Patient and public involvement

Early findings were presented to members of the Research Advisory Panel within the NIHR Applied Research Collaboration North Thames. The direction of the study and findings were refined following the panel’s comments.

## Results

### Sample characteristics

The characteristics of the 162 HEIs considered in the analysis are summarised in table 1. HEIs are fairly equally spread across the different regions, with the highest number of institutions in London (23.5%). The sample includes all 24 Russell Group universities, constituting less than a fifth (14.8%) of the total sample. Under a quarter (21.0%) of the institutions considered opted out of providing information about non-academic staff from 2019/2020 onwards, and of these, nearly a tenth (8.8%) are part of the Russell Group. Furthermore, more than half (61.1%) of all HEIs are members of the Athena SWAN Charter, with the majority (74.7%) holding a Bronze award. No HEIs hold a Gold award but for some (8.0%) it was not possible to establish their membership status. Over two-thirds (71.6%) are part of the Disability Confident Employer Scheme. Of these, a handful (8.6%) have reached Level 3 (Disability Confident Leader) while most (65.5%) are at Level 2 (Disability Confident Employer). Less than a third (28.4%) of HEIs are not members of the Disability Confident Scheme. Notably, 58.1% of Athena SWAN Bronze award holders are likely to have reached Level 2 on the Disability Confident Employer Scheme (results not reported in detail but available on request).

**Table 1:** Summary statistics

Region	***N***	%
North	32	19.8
Midlands	31	19.1
London	38	23.5
South	32	19.8
Northern Ireland/Scotland/Wales	29	17.9
**Optout**		
No	128	79.0
Yes	34	21.0
**Russell Group**		
No	138	85.2
Yes	24	14.8
**Athena SWAN**		
No award	50	30.9
Bronze	74	45.7
Silver	25	15.4
Gold	–	–
Missing	13	8.0
**Disability Confident Employer**		
Non-member	46	28.4
Committed (Level 1)	30	18.5
Employer (Level 2)	76	46.9
Leader (Level 3)	10	6.2
**Report availability**		
No	35	21.6
Yes	127	78.4
**Report detail (grouped)**		
No detail	17	13.4
Little detail	74	58.3
Some detail	36	28.3
No report	35	21.6
**Report detail type**		
Trends	88	69.3
Staff group	57	44.9
Disability type	31	24.4
Grade	21	16.5
Employment mode	16	12.6
Contract type	15	11.8
Faculty/service area	11	8.7

### EDI report availability

With regards to EDI reports, overall, under a quarter (21.6%) of HEIs do not have a publicly available report online (see table 1). When differentiating report availability by EDI charters, the picture is different depending on the charter considered (see table 2). On the one hand, there is a 21.9% statistically significant difference (at 1% level) between award and non-award Athena SWAN holders—specifically, 85.9% of award holders have an open EDI report compared with 64.0% of non-award holders. Looking at the different award levels, a plethora of HEIs with a Bronze or Silver award have a publicly available report (87.8% and 80.0%, respectively; results available on request). On the other hand, when focusing on Disability Confident, members of the scheme are slightly more likely to have a report openly available than non-members (80.2% vs 73.9%, respectively), but the difference is not statistically significant. Between-level differences in report availability are also small and insignificant—for instance, 76.7% of HEIs at Level 1 have a publicly available report versus 80.0% of HEIs at Level 3.

**Table 2:** EDI report availability by EDI charter membership

	Athena SWAN member			Disability Confident member	
	**No (1**)	**Yes (2**)	**Missing (3**)	**No (4**)	**Yes (5**)
**Report availability**					
Mean (SD)	0.640 (0.485)	0.859 (0.350)	0.769 (0.439)	0.739 (0.444)	0.802 (0.400)
*N*	50	99	13	46	116
	(1)-(2)	–	–	(4)-(5)	–
Difference (SE)	−0.219* (0.069)	–	–	−0.063† (0.072)	–

* *p* < 0.01

† not significant

EDIequality, diversity and inclusionSDstandard deviationSEstandard error

**Figure 1:**
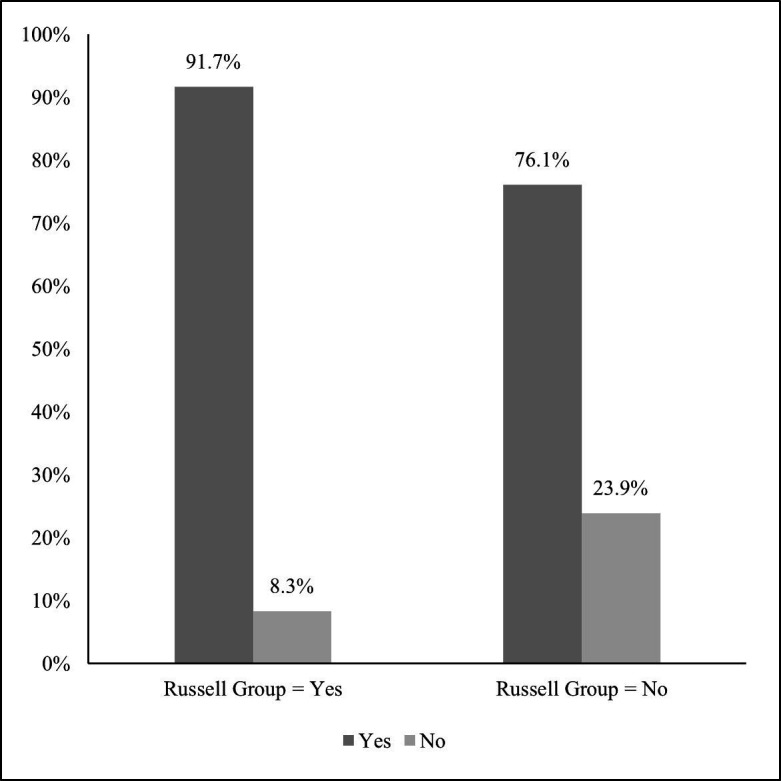
Equality, diversity and inclusion (EDI) report availability by Russell Group membership. Note: The difference between Russell Group and non-Russell Group universities is statistically significant at 10% level.

When considering differences by institution type, Russell Group universities are more likely to have a report available (91.7% vs 76.1%, respectively), and the difference is marginally statistically significant at 10% level (see figure 1 for more details). For opt out, HEIs not opting out of providing information about non-academic staff from 2019/2020 onwards are more likely to have a publicly available EDI report than those opting out (82.0% vs 64.7%, respectively; see online supplemental figure 1 for more details), and the difference is statistically significant at 5% level.

**Figure 2:**
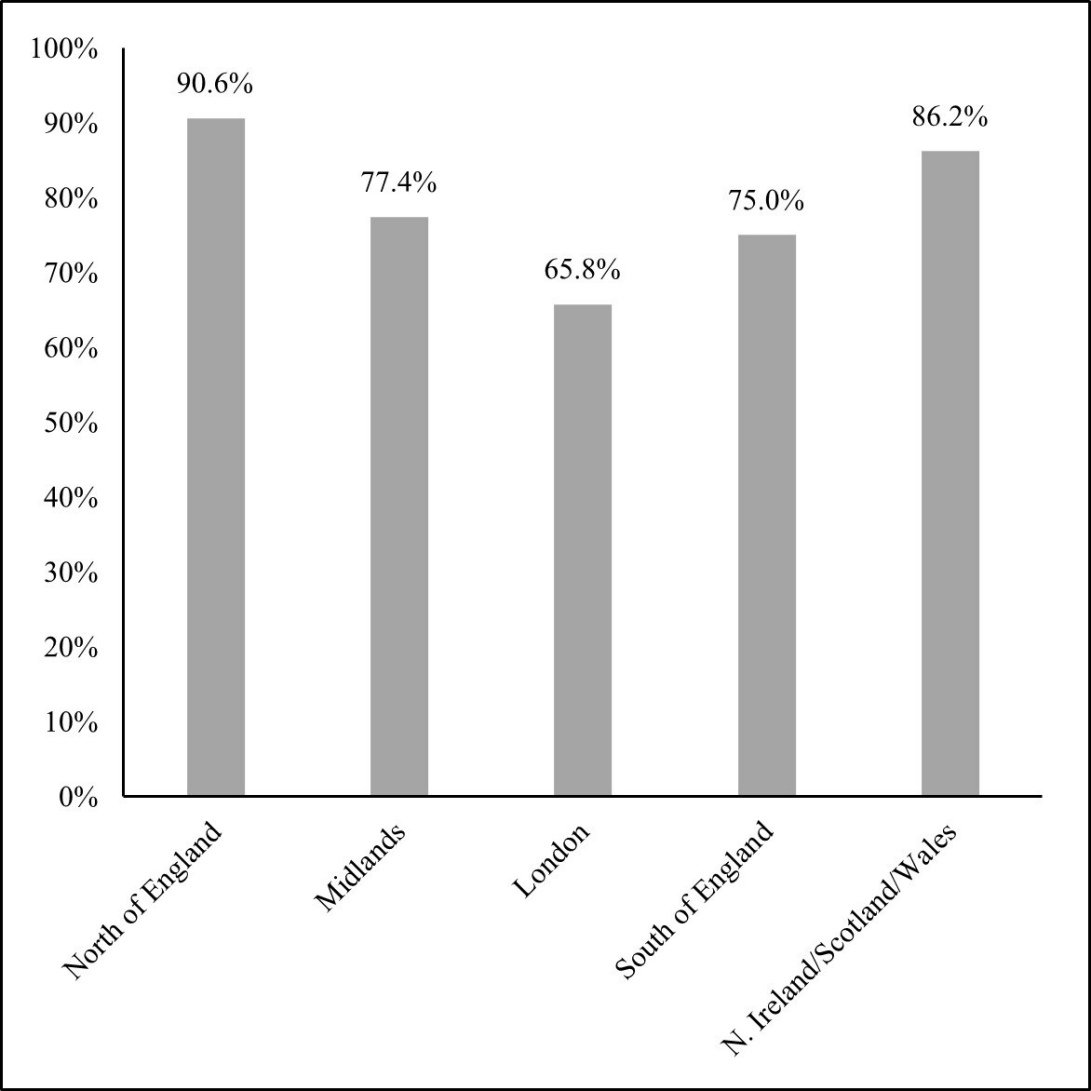
Equality, diversity and inclusion (EDI) report availability by region. Note: The cross-regional difference is statistically significant at 5% level when comparing London with either the North of England, or Northern Ireland, Scotland and Wales.

At the regional level, EDI report availability is highest for HEIs in the North of England (90.6%) followed by Northern Ireland, Scotland and Wales (86.2%). Institutions in the Midlands and South of England have similar report availability (77.4% and 75.0%, respectively) while the lowest percentage is found to be in London (65.8%) (see figure 2). The difference in report availability when comparing London with the remaining regions is statistically significant (at 5% level) for the North of England, and Northern Ireland, Scotland and Wales. The limited report availability in London may be linked with the location of institutions and/or HEIs’ staff size (ie, several smaller institutions in the same region). Indeed, when looking at staff size, the average number of staff is lowest, on average, in London compared with institutions in the remaining regions (for more information, see online supplemental material), and this is irrespective of opting out or not of submitting information for non-academic staff from 2019/2020 onwards.

### EDI report detail

**Figure 3:**
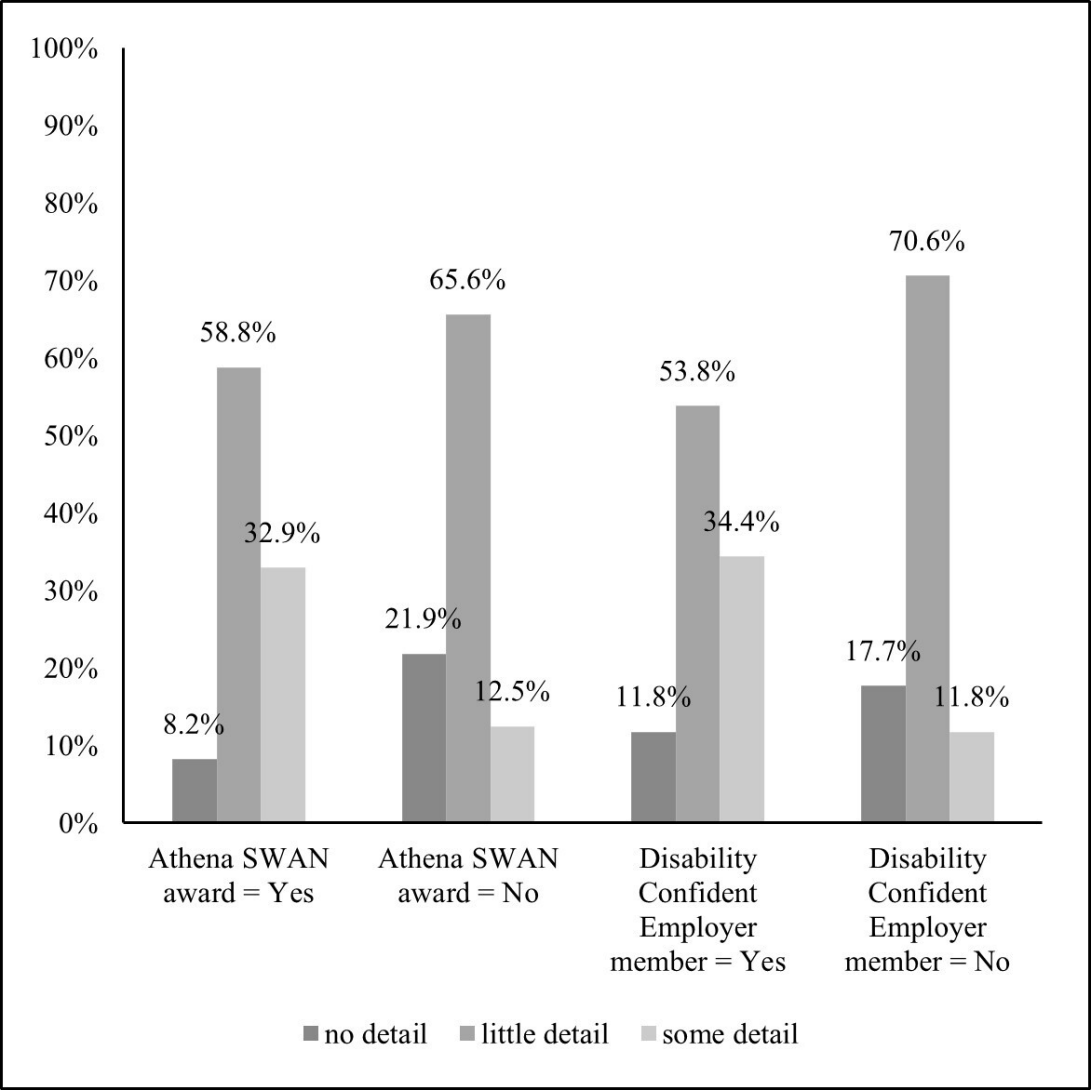
Equality, diversity and inclusion (EDI) report detail by EDI charter membership—Athena SWAN and Disability Confident Employer. Note: Athena SWAN award: The difference for ‘no detail’ is statistically significant at 5% level. The difference for ‘little detail’ is not statistically significant. The difference for ‘some detail’ is statistically significant at 5% level. Disability Confident Employer member: The difference for ‘no detail’ is not statistically significant. The difference for ‘little detail’ is statistically significant at 10% level. The difference for ‘some detail’ is statistically significant at 5% level.

The level of detail with regards to staff disability varies in the sample. More than half (58.3%) of the institutions provide up to two additional characteristics (ie, little detail) other than current staff disability prevalence, and just under a third (28.3%) more than two extra characteristics (ie, some detail). Staff disability prevalence comparisons with previous years (labelled here as trends) is the most frequent (69.3%) additional feature among institutions, followed by staff group (44.9%), disability type (24.4%) and (pay) grade (16.5%). The least frequent feature is faculty/service area (8.7%) (see table 1). It is worth noting that the level of detail also varies by EDI charter; on average, Athena SWAN award holders are twice as likely to provide ‘some detail’ as their non-member counterparts (32.9% vs 12.5%, respectively), and the difference is statistically significant at 5% level (see figure 3). In the same vein, Disability Confident institutions are twice as likely to provide ‘some detail’ as those that are not part of the scheme (34.4% vs 11.8%, respectively), which is also statistically significant at 5% level (see figure 3).

## Discussion and conclusions

This analysis adds to the literature on staff disability within higher education in the UK, providing detail on the extent of EDI reporting and the use of equality charters, specifically Athena SWAN and the Disability Confident Employer Scheme. Limited research to date examining the use and impact of such charters has primarily been qualitative (Bhopal and Henderson 2019) whereas this study provides a cross-sector quantitative analysis of EDI reporting in practice.

Open reporting of EDI data enables comparisons to be drawn across the sector, highlighting differences between sizes and types of institutions for instance, as well as allowing analysis of change over time within institutions and within higher education more broadly. One area of comparison can be seen with the higher level of EDI reporting among Russell Group universities compared with non-Russell Group universities. This may reflect the size and availability of resources at these research-intensive institutions. Moreover, those HEIs that opt out of providing data on non-academic staff are also less likely to have an EDI report. This may be related to issues with identification of individuals but may also reflect institutional cultures where staff feel less comfortable reporting on disability or other protected characteristics which may relate to lack of transparency in EDI reporting. EDI data are predicated on staff feeling willing to disclose information about their personal characteristics, which is often cited as an issue for staff with disabilities in the literature (Brewster *et al* 2017). Our analysis showed that HEIs in London are less likely to have an EDI report publicly available. We found that these are likely to be smaller institutions, and institutional size may play a role in the lack of reporting, with smaller, potentially less well-resourced institutions having smaller EDI teams. Contributing to this could also be the location of the institutions per se.

Our analysis further explored the level of detail in terms of staff disability that the EDI reports provide. Institutions signed up to the two charters considered here are more likely to provide a greater level of detail on staff disability in their reports than non-members, possibly reflecting a deeper engagement with issues of staff disability. This also suggests that charters remain an important way to motivate HEIs to both monitor and report EDI data, which is a vital step preceding action planning for interventions. Interestingly, more institutions are members of the Disability Confident Scheme than Athena SWAN award holders, which may be due to the more stringent requirements present for the Athena SWAN Charter (Advance HE n.d.-a). However, it is rare for HEIs to be awarded the highest level of either of these charters. No institutions have achieved Athena SWAN Gold status while the highest Disability Confident level (Leader), which requires external validation of an organisation’s activities, has been achieved by only 6.2% of the institutions considered. This suggests that much work is still needed to make HEIs more equitable.

It is important to highlight that as a national government initiative, the Disability Confident Scheme is not tailored to higher education, unlike Athena SWAN. It outlines steps employers can take to be classed as Disability Confident, for instance, committing to interviewing applicants with disability who meet the minimum criteria for a role. However, the actions required to improve participation and retention among workers with disabilities may not address the myriad issues that academics with disabilities, in particular, report facing within the higher education system, such as a culture of elitism and productivity that does not make allowance for an individual’s abilities.

There are several limitations to note in this study. Much of the literature on staff with disabilities in academia focuses on academic staff. This demarcation is reflected in this study, with 21% of institutions choosing to opt out of providing data on non-academic staff, limiting the conclusions that can be drawn on this large staff group. Adding to this, there is no information available for smaller HEIs (those with fewer than 150 employees), which are exempt from the PSED. Similarly, private/for-profit HEIs were excluded, although arguably these constitute a proportion of the UK higher education sector (Shury *et al* 2016). Furthermore, although EDI charters address individual protected characteristics such as gender, disability or race, these silos do not reflect the reality at an individual level. Each person has multiple identities which intersect, and may intersect to compound the effect of each individual structural barrier for people who experience multiple levels of marginalisation (eg, a pregnant black woman with disability vs a white man without disability).

This study highlights that challenges remain to gain a clear picture of staff disability within the higher education workforce. Although HEIs are required to gather data under the PSED, there is a lack of uniformity and transparency in how the data are collected and interpreted, which can limit the ability to develop meaningful EDI interventions for understudied and structurally disadvantaged staff with disabilities, but also to address inequities across institutions more broadly. Future directions for research could include linking institutions’ public EDI data to their mission statements to assess whether HEIs are meeting their own targets; analysis of whether larger HEIs have higher disability prevalence; and investigation of whether the level of detail in EDI reports changes over time as EDI practices become more embedded.

## supplementary material

10.1136/medhum-2024-012892 Supplementary file 1

## Data Availability

Data are available upon reasonable request.
