# Supplementary material for: Staff disability data in UK higher education: Evidence from EDI reports
Source: Med Humanit. 2024 Jun 4;50(3):e012892. doi: 10.1136/medhum-2024-012892 (PMC11503180; doi:10.1136/medhum-2024-012892)
Supplement: Supplementary file 1 [file medhum-50-3-s001.pdf]

Supplementary material

Supplemental Table 1

Data fields and definitions

Field name	Description	Categories
Opt-out	Opted out from returning information about non-academic staff in 2019/20 onwards	0 = No<br>1 = Yes
Region	Region the institution belongs to (collapsed)	1 = North of England<br>2 = Midlands<br>3 = London<br>4 = South of England<br>5 = Northern Ireland/Scotland/Wales
Report year	Year mentioned in the cover of the EDI report. If not mentioned, it was identified on a case-by-case basis from the main text.	1 = Before 2018/19<br>2 = 2018/19<br>3 = 2019/20<br>4 = 2020/21<br>5 = 2021/22
Report frequency	How many EDI reports in each institution are publicly available	0,…,13
Disability status	This is a derived variable based on the definition/labelling used by each institution. Notes: 'Declared disability' or 'Disabled' coded as Dis. 'Declared non-disabled' or 'No known disability' typically coded as No dis. 'Unknown' typically coded as NK	1 = Dis only<br>2 = Dis; No dis OR Dis; No dis/NK<br>3 = Dis; No dis; Other
Disability type	Similar to the HESA grouping (https://www.hesa.ac.uk/collection/c20025/a/disable) or in a collapsed form	0 = No<br>1 = Yes
Trends	If there is any comparison with previous years in the text or graphically or in the tables	0 = No<br>1 = Yes

Trends frequency	How many years current prevalence rates are being compared with	0,…,11
Faculty/service area	Staff disability by: Faculty/discipline area	0 = No<br>1 = Yes
Staff group	Staff disability by: Academic, non-academic, professional staff. This can also refer to Teaching & Research, Research only, Teaching only, etc.	0 = No<br>1 = Yes
Contract type	Staff disability by: Casual, fixed-term, permanent, etc.	0 = No<br>1 = Yes
Employment mode	Staff disability by: Full-time, part-time	0 = No<br>1 = Yes
Grade	Staff disability by: Grade/pay level	0 = No<br>1 = Yes
Russell Group	Member of Russell Group	0 = No<br>1 = Yes
Athena SWAN	Member of the Athena SWAN Charter (https://www.advance-he.ac.uk/equality-charters/athena-swan-charter/members#Members – accessed 25 January 2023)	0 = Not a member<br>1 = Bronze<br>2 = Silver<br>3 = Gold
Disability Confident Employer	Member of the Disability Confident Employer Scheme (https://assets.publishing.service.gov.uk/government/uploads/system/uploads/attachment_data/file/1127368/disability-confident-list-of-employers.csv/preview – accessed 25 January 2023)	0 = Not a member<br>1 = Committed<br>2 = Employer<br>3 = Leader

Supplemental Table 2

Staff size by region and opt-out option

	**Opted-out†**	**Not opted-out**
**North**	1,137 (1,126)	2,826 (2,201)
**Midlands**	1,763 (1,602)	2,750 (2,756)
**London**	562 (378)	2,437 (2,436)
**South**	1,554 (2,696)	3,444 (3,162)
**Northern Ireland/Scotland/Wales**	1,440 (N/A)	2,778 (3,222)

Note. Means are reported and standard deviations in parentheses. N/A, not available. †Staff size for opt-out reflects academic staff only. For opt-out institutions with approximately 100 academic staff, we set 40% of academic staff as the minimum for non-academic staff, based on the average from non-opt-out institutions.

Supplemental Figure 1

EDI report availability by opt-out option

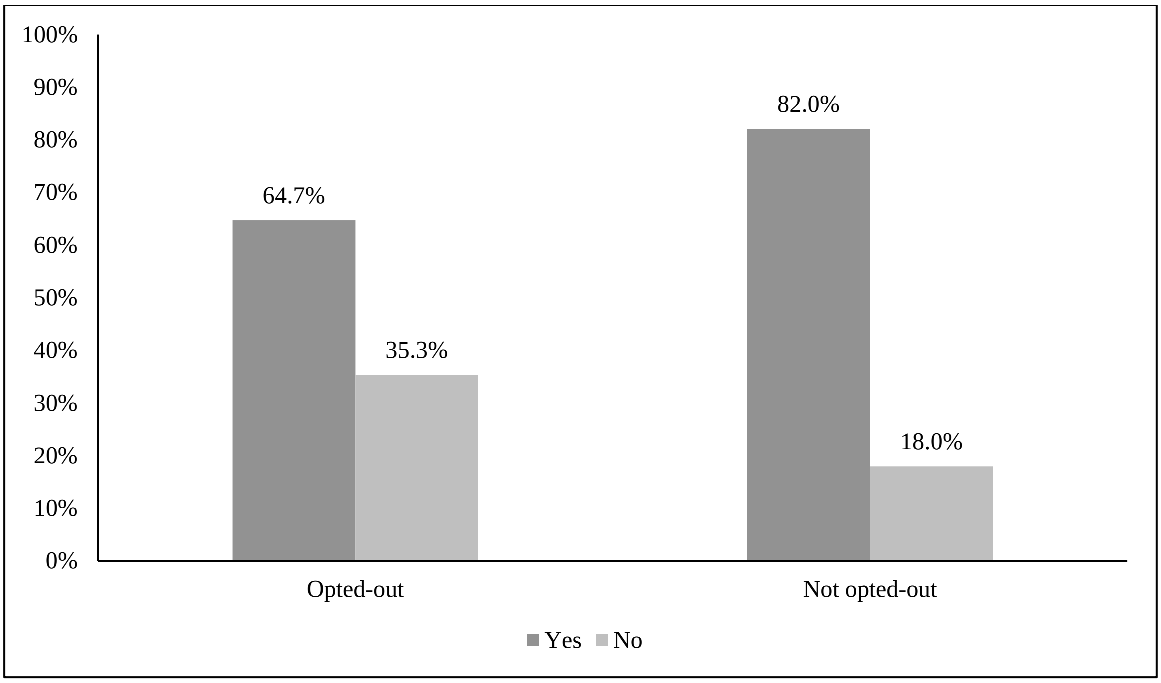

Note. The difference between universities that opted-out and those that did not opt-out is statistically significant at 5% level.
